# The neuroprotective steroid progesterone promotes mitochondrial uncoupling, reduces cytosolic calcium and augments stress resistance in yeast cells

**DOI:** 10.15698/mic2017.06.577

**Published:** 2017-05-31

**Authors:** Slaven Stekovic, Christoph Ruckenstuhl, Philipp Royer, Christof Winkler-Hermaden, Didac Carmona-Gutierrez, Kai-Uwe Fröhlich, Guido Kroemer, Frank Madeo

**Affiliations:** 1 Institute of Molecular Biosciences, NAWI Graz, University of Graz, 8010 Graz, Austria.; 2 BioTechMed Graz, Austria.; 3 Equipe 11 labellisée par la Ligue contre le Cancer, Centre de Recherche des Cordeliers, Paris, France.; 4 INSERM, U1138, Paris, France.; 5 Université Paris Descartes, Sorbonne Paris Cité, Paris, France.; 6 Cell Biology & Metabolomics Platforms, Gustave Roussy Comprehensive Cancer Center, Villejuif, France.; 7 Pôle de Biologie, Hôpital Européen Georges Pompidou, AP‐HP, Paris, France.; 8 Karolinska Institute, Department of Women's and Children's Health, Karolinska University Hospital, 17176 Stockholm, Sweden,

**Keywords:** TBI, traumatic brain injury, cell protection, cell stress, cell death, neuroprotection, progesterone, mitochondrial uncoupling

## Abstract

The steroid hormone progesterone is not only a crucial sex hormone, but also serves as a neurosteroid, thus playing an important role in brain function. Epidemiological data suggest that progesterone improves the recovery of patients after traumatic brain injury. Brain injuries are often connected to elevated calcium spikes, reactive oxygen species (ROS) and programmed cell death affecting neurons. Here, we establish a yeast model to study progesterone-mediated cytoprotection. External supply of progesterone protected yeast cells from apoptosis-inducing stress stimuli and resulted in elevated mitochondrial oxygen uptake accompanied by a drop in ROS generation and ATP levels during chronological aging. In addition, cellular Ca^2+^ concentrations were reduced upon progesterone treatment, and this effect occurred independently of known Ca^2+^ transporters and mitochondrial respiration. All effects were also independent of Dap1, the yeast orthologue of the progesterone receptor. Altogether, our observations provide new insights into the cytoprotective effects of progesterone.

## INTRODUCTION

Progesterone is a sterol-derived hormone that is crucial for female reproductive capacity and plays major regulatory roles in the monthly menstrual cycle and upon conception as well as during pregnancy and embryogenesis. In addition, it also serves as a neurosteroid, thus playing an important role in brain function in both sexes 1. For instance, progesterone inhibits the neuronal nicotinic acetylcholine receptor and stimulates the synthesis of myelin proteins 1. Of note, progesterone has been linked to the gender-specific risk and outcome of brain injuries that is more favorable for females 2. Interestingly, preclinical data strongly suggest that (high doses of) progesterone may positively affect recovery from traumatic brain injury (TBI) in model organisms 3,4,5,6,7, if administered before or shortly after TBI. Two clinical studies could confirm a neuroprotective effect of progesterone when administered shortly after TBI 8,9, while some more recent clinical data seem to disprove this hypothesis 10,11,12. Therefore, it remains an open question if progesterone affects the recovery and survival after TBI in humans and to which extent it promotes cellular restauration.

In order to investigate the cytoprotective potential of progesterone, we took advantage of *Saccharomyces cerevisiae*, knowing that this organism has repeatedly been shown to be suitable for mechanistic studies of programmed cell death (PCD) 13,14,15,16,17,18,19. Yeast is especially useful as a model to study neuroprotection at the cellular level 20,21,22,23,24,25,26,27. Here, we describe the positive impact of progesterone on several parameters of cellular physiology. Importantly, our results also suggest a possible receptor-independent mechanism for these effects, since deletion of *DAP1* - a heme-binding protein related to the mammalian membrane progesterone receptor - did not alter susceptibility towards progesterone treatment. Altogether, we reveal that progesterone exerts potent cytoprotective effects in yeast.

## RESULTS

### Progesterone increases stress tolerance

**Figure 1:**
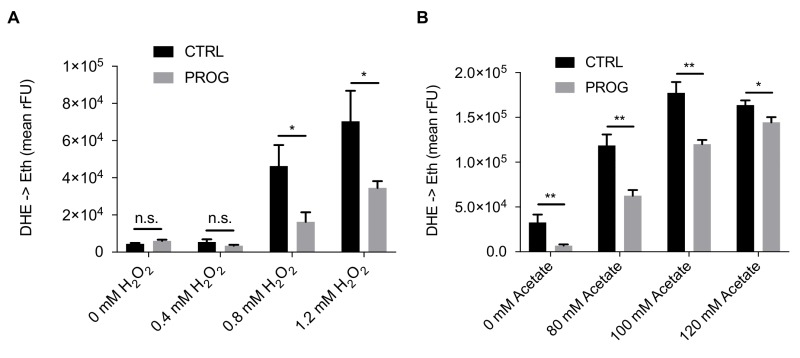
FIGURE 1: Progesterone treatment increases resistance of wildtype yeast to external stressors. ROS accumulation in yeast cells treated with progesterone (10 µg/ml) or left untreated as shown by the DHE to ethidium turnover rate upon hydrogen peroxide **(A)** or acetate **(B)** challenge during logarithmic phase. All data represent mean values (n = 3 ± SEM). Statistical analysis was conducted using non-paired Student’s t-test. * = p<0.05; ** = p<0.01; *** = p<0.001; n.s. = non-significant, Prog = progesterone, ctrl = control.

**Figure 2:**
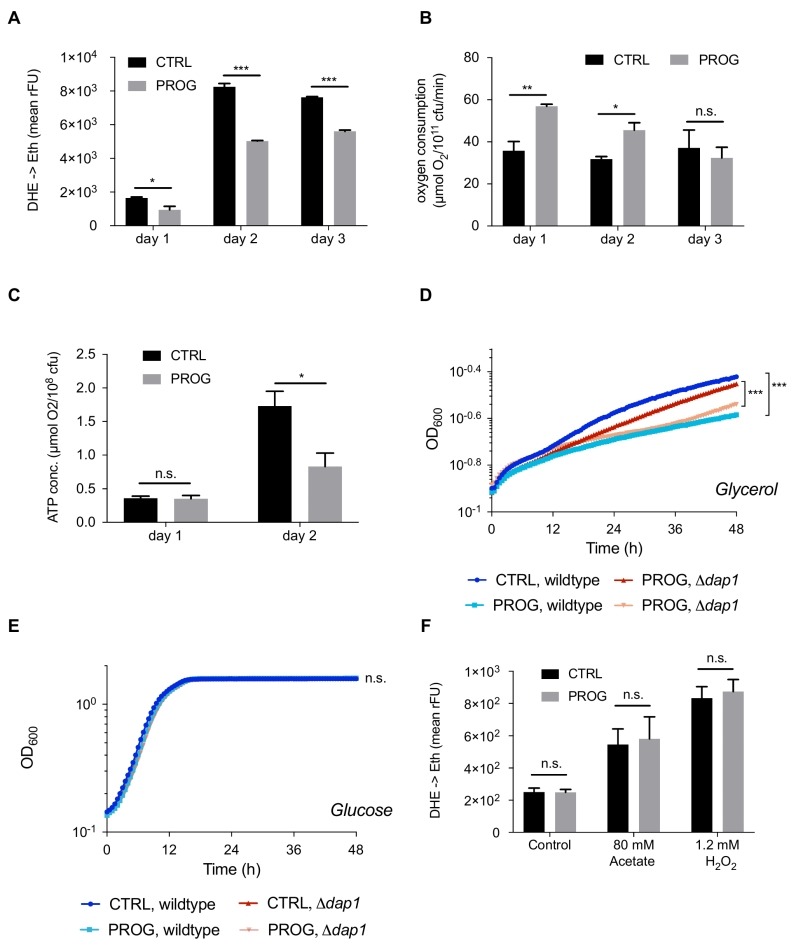
FIGURE 2: Progesterone impacts energy metabolism and reduces oxygen stress accumulation in wildtype yeast. Wildtype yeast were treated with 10 µg/ml progesterone and assayed for **(A)** ROS accumulation via DHE to ethidium turnover, **(B)** oxygen consumption via respirometry, and **(C)** ATP production. Growth curves of wildtype as well as ∆*dap1* strains, with or without progesterone treatment, on glycerol (respiratory carbon source) **(D)** and glucose (fermentative carbon source) media **(E)**. ROS accumulation in rho^0^ yeast cells +/- progesterone (10 µg/ml) treated or untreated with H_2_O_2_ or acetate during logarithmic phase **(F)**. All data represent mean values (n = 3-5 ± SEM). Statistical analysis was conducted using non-paired Student t-test (A-C, F) or using a two-way repeated measurement ANOVA and multiple comparison post-hoc Tukey’s test (D, E). * = p<0.05; ** = p<0.01; *** = p<0.001; n.s. = non-significant. ROS = reactive oxygen species, rFU = relative fluorescence units, Prog = progesterone, ctrl = control.

Traumatic brain injury is connected to elevated PCD and ROS accumulation in the brain tissue 28,29. Therefore, we tested if progesterone would render yeast cells less susceptible towards different stressors that are connected to an increase in ROS production. Upon addition of progesterone, wild type yeast cultures treated with H_2_O_2_ or acetate, which are both well-known PCD inducers in yeast 14,30,31,32,33,34, showed reduced ROS accumulation as measured by the ROS-driven conversion of dihydroethidium (DHE) to fluorescent ethidium (Figure 1A and B). Furthermore, under physiological culture conditions, in the absence of PCD inducers, progesterone significantly reduced ROS levels as compared to the untreated control (Figure 2A). Altogether, progesterone dampens ROS production in yeast, both in normal culture conditions and in the presence of external stress factors.

### Progesterone impacts mitochondria by acting as a mild respiration-uncoupler

To further explore the mechanisms underlying progesterone cytoprotection, we next examined the physiology of mitochondria, since these organelles constitute one of the main sources of ROS 35,36,37,38. Interestingly, while O_2_ consumption was significantly enhanced during progesterone treatment, ATP levels were reduced (Figure 2B and C). Altogether, this indicates an uncoupling phenotype with diminished oxidative phosphorylation. Accordingly, we observed reduced growth of wild type yeast upon progesterone treatment on a non-fermentable carbon source (glycerol), while no changes were detected on a fermentable carbon source (glucose) (Figure 2D and E). Importantly, this effect was also observed in a mutant strain lacking the heme-binding protein Dap1, which is the sole yeast orthologue of the human progesterone receptor (Figure 2D and E) 39. Furthermore, we could demonstrate that stress protection by progesterone is respiration-dependent, since progesterone treatment did not confer stress resistance in respiration-deficient rho^0^ cells (Figure 2F). Altogether, it appears that progesterone impacts yeast mitochondrial respiration in a receptor-independent fashion.

### Progesterone administration diminishes cytosolic Ca^2+^ concentrations both under physiological as well as under high calcium conditions

**Figure 3:**
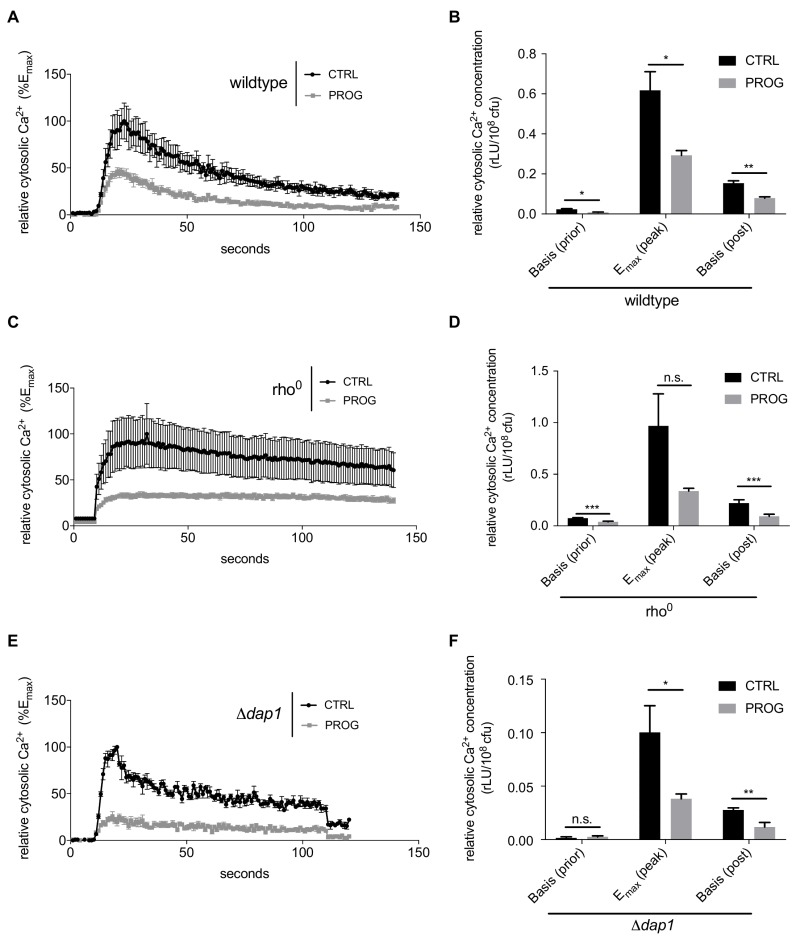
FIGURE 3: Cellular Ca^2+^ homeostasis is modulated by progesterone treatment in yeast. Cells were treated with progesterone (10 µg/ml) and challenged with high doses of Ca^2+^ (150 mM). Intake of Ca^2+^ as well as Ca^2+^-clearance in the cytosol to its basal level were measured in wild type **(A,B)**, a *DAP1*-deletion strain **(C,D)**, as well as in a rho^0^ strain, incapable of mitochondrial respiration **(E,F)**. Data are shown as mean values of at least three replicates including the standard error of the mean. Statistical analysis was conducted using non-paired Student’s t-test. * = p<0.05; ** = p<0.01; *** = p<0.001; n.s. = non-significant. E_max_ = global maximum of the respective ethanol-treated control; rLU = relative luminiscence units, Prog = progesterone, ctrl = control.

Next we investigated progesterone effects on Ca^2+^ homeostasis, knowing that mitochondria are one of the organelles responsible for buffering cytosolic Ca^2+^ under normal conditions 40. Importantly, TBI, stroke, and even some forms of dementia cause Ca^2+^ accumulation in the cytosol of neurons followed by cell death and neurodegeneration 41. Thus, we examined the capacity of yeast cells to process Ca^2+^ uptake under the influence of progesterone. Specifically, wild type yeast cell cultures were challenged with 150 mM CaCl_2_ and transient concentrations of cytoplasmic Ca^2+^ levels ([Ca^2+^]cyt) / responses were monitored. Progesterone caused a significantly reduced Ca^2+^ uptake capacity alongside with a faster reduction of cytoplasmic Ca^2+^ levels (Figure 3A and B). Of note, basal Ca^2+^ levels before and after the Ca^2+^ pulse were already lowered when cells were treated with progesterone (Figure 3B). However, mitochondrial respiration was not involved in this phenotype, since progesterone treatment continued to affect basic cytosolic Ca^2+^ levels in respiration-deficient rho^0^ cells (Figure 3C and D).

To further investigate the observed phenotypes, we tested single-gene deletion mutants of all currently known Ca^2+^ channels/transporters in yeast, including the cytoplasmic membrane transporters Cch1 and Mid 1, the organelle transporters Vcx1, Pmr1, Cod1, Yvc1, and Pmc1 as well as Emc7, an ER protein associated to Ca^2+^ homeostasis. Although Ca^2+^ uptake and clearance was influenced by some of these gene deletion, all mutants continued to exhibit significantly reduced Ca^2+^ uptake when treated with progesterone (compare Supplemental Figure 1A-G to H). Thus, the effects observed in wild type cells could not be reversed by single gene deletions in any of these transporters. Similarly, the effects of progesterone treatment on Ca^2+^ homeostasis/uptake were independent of the mammalian membrane progesterone receptor homolog Dap1 (Figure 3E and F). Taken together, progesterone seems to influence Ca^2+^ homeostasis/uptake in a general manner, independently from known Ca^2+^ transporters and respiration capacity.

### DISCUSSION

Here, we establish *S. cerevisiae* as a model to investigate cytoprotection by progesterone. We observed that progesterone increased stress tolerance of yeast to the well-known PCD inducers H_2_O_2_ and acetate 14,30,31,32,33,34 as well as under physiological (control) conditions. Interestingly, progesterone treatment led to a mild uncoupling phenotype with higher O_2_ consumption (+50%) but lower ATP levels (-50%), arguing for a mitochondrial uncoupling effect. Indeed, growth on the non-fermentable carbon source glycerol was diminished in the presence of progesterone. Notably, mild uncoupling induced by chemical substances (such as dinitrophenol), caloric restriction or ectopic expression of mammalian uncoupling proteins in yeast - *S. cerevisiae* does not possess any known uncoupling proteins 42 - is known to increase lifespan 43,44,45. Similarly, in mammalian aging cells, changes in mitochondrial energy metabolism caused by mitochondrial uncoupling seem to improve cellular fitness 46. Progesterone treatment of human cells has been demonstrated to strongly increase the levels of mRNAs coding for uncoupling proteins 47. Increased O_2_ consumption with decreased ^32^P uptake (as a parameter for ATP production) has been reported for isolated rat mitochondria treated with progesterone 48. Collectively, our data combined with those reported in the literature highlight the possibility to investigate progesterone-mediated effects in the yeast model. The uncoupling aspect of progesterone, in fact, could represent one of the mechanisms of neuroprotection conferred by this steroid. In fact, the stress tolerance of a respiration-deficient rho^0^ strain was not influenced by progesterone treatment.

Progesterone had major effects on Ca^2+^ homeostasis and, in particular, on Ca^2+^ susceptibility/uptake. However, we could not identify any single Ca^2+^ channel in yeast that would influence these effects. However, we cannot exclude that yet unidentified Ca^2+^ channels or a combinations of known Ca^2+^ channels mediate these effects 49. Another possible mode of action of progesterone on Ca^2+^ homeostasis could reside in its direct interaction with biological membranes. Since the chemical structure of progesterone shows four-ring as well as hydrophobic backbone and polar groups at both ends of the molecule, it could directly interact with cellular and mitochondrial membranes 50 and possibly influence their permeability towards inorganic cations (e.g. Ca^2+^, H^+^). This mode of action could connect our observations of mitochondrial uncoupling and modulation of Ca^2+^ homeostasis. Of note, a progesterone-treated rho^0^ strain still showed Ca^2+^ effects but no enhanced stress tolerance, suggesting that altered Ca^2+^ homeostasis may lie upstream of mitochondrial uncoupling. However, these mechanistic hypotheses remain to be empirically tested.

Certainly, the putative relevance of the herein described progesterone effects for TBI pathology remains to be explored. In some mammalian cell types, progesterone leads to a significant increase of intracellular Ca^2+^ 51,52, partly by activating protein kinase C 53 and depleting endocannabinoids by activating α/β hydrolase domain-containing protein 2 (ABHD2) 54. However, in other cell types, progesterone withdrawal leads to an increased level of cytosolic Ca^2+^ 55. While progesterone was not able to reduce estrogen-induced Ca^2+^ uptake in the rabbit myometrial smooth muscle cells, it increased the accumulation of Ca^2+^ in mitochondria 55. This suggests that progesterone withdrawal reduces both myometrial cytosolic Ca^2+^ levels as well as the capacity of these cells to accumulate Ca^2+^in different cellular compartments. Similar effects were reported for other types of smooth muscles 56,57 and are believed to be caused by regulation of the inward current through L-type Ca^2+^ channels 56,58. In neurons, the influence on Ca^2+^ signaling and the following inhibition of excitotoxic neuron death seem to be the neuroprotective mechanism induced by acute administration of progesterone after various neuronal injuries 59,60,61. Indeed, progesterone might mediate broad neuroprotective effects, not only in the context of TBI but also in other pathologies 62,63.

The role of progesterone in the pathological development of TBI has been well described in recent years. It has been shown that progesterone improves the function of the blood-brain-barrier after TBI 64. Progesterone also increases the level of circulating endothelial cells, which in turn improves neovascularization and vascular remodeling in the brain 65. Furthermore, progesterone treatment reduces neuroinflammation and oxidative stress 66 as it improves remyelination and functional recovery 63.

Interestingly, the intracellular effects exerted by progesterone in our model - reduced intracellular Ca^2+^ levels, uncoupled mitochondria and ROS reduction - were not lost when the sole possible yeast orthologue of the human progesterone receptor was removed from the system. This suggests that progesterone mediates its broad cytoprotective effects through other proteins than steroid receptors or perhaps with cellular membrane lipids. We surmise that yeast constitutes an ideal platform for exploring these effects in further detail.

### MATERIALS AND METHODS

#### Growth conditions

*S. cerevisiae *strains (Table 1) were inoculated to 5*10^5^ (for growth curve OD_600_ of 0.05) cells in SC medium containing 0.17% yeast nitrogen base (BD Diagnostics; without ammonium sulfate and amino acids), 0.5% (NH_4_)_2_SO_4_ , 30 mg/L of all amino acids (except 80 mg/L histidine and 200 mg/L leucine), 30 mg/L adenine, and 320 mg/L uracil with 2% glucose (SCD) or alternatively with 3% glycerol (SCGly), w/o treatment with progesterone (10 µg/ml; Sigma Aldrich, Catalogue Nr. P0130). Controls were treated with respective solvent (EtOH). Where indicated, stress (acetate or H_2_O_2_) was inflicted as described previously in mid-log phase (~ 6h of growth, culture density 2-4*10^6^ cells/ml). Due to the inherent reduced respiration-rate of BY4741 strains, TB50a strains were used for respiration-related experiments. *DAP1* deletion was carried out by classical homologous recombination 67,68.

**Table 1:** Strains used in this study.

**Strain**	**Genotype**	**Reference**
TB50a wild type	*MATa; leu2-3,112 ura3-52 trp1 his3 rme1 HMLa*	69
TB50a *∆dap1*	*MATa; leu2-3,112 ura3-52 trp1 his3 rme1 HMLa dap1::kanMX*	This study
BY4741 wild type	*MATa his3Δ1 leu2Δ0 met15Δ0 ura3Δ0*	Euroscarf
BY4741 *∆dap1*	*MATa his3Δ1 leu2Δ0 met15Δ0 ura3Δ0* *dap1::kanMX*	Euroscarf
BY4741 *∆cch1*	*MATa his3Δ1 leu2Δ0 met15Δ0 ura3Δ0 cch1::kanMX*	Euroscarf
BY4741 *∆mid1*	*MATa his3Δ1 leu2Δ0 met15Δ0 ura3Δ0 mid1::kanMX*	Euroscarf
BY4741 *∆vcx1*	*MATa his3Δ1 leu2Δ0 met15Δ0 ura3Δ0 vcx1::kanMX*	Euroscarf
BY4741 *∆pmr1*	*MATa his3Δ1 leu2Δ0 met15Δ0 ura3Δ0 pmr1::kanMX*	Euroscarf
BY4741 *∆cod1*	*MATa his3Δ1 leu2Δ0 met15Δ0 ura3Δ0 cod1::kanMX*	Euroscarf
BY4741 *∆yvc1*	*MATa his3Δ1 leu2Δ0 met15Δ0 ura3Δ0 yvc1::kanMX*	Euroscarf
BY4741 *∆pmc1*	*MATa his3Δ1 leu2Δ0 met15Δ0 ura3Δ0 pmc1::kanMX*	Euroscarf
BY4741 *∆emc7*	*MATa his3Δ1 leu2Δ0 met15Δ0 ura3Δ0 emc7::kanMX*	Euroscarf

#### Growth curve

Cells from ONC in SCD media were inoculated to an OD_600_ of 0.05 in SCD media and SCGly media with or without 10 µg/ml progesterone addition. Untreated cultures were supplemented with 0.1% EtOH for solvent control. To obtain growth curves, 300 µl of respective cultures per well were transferred into Honeycomb^®^ plates, and measured with Bioscreen C MBR system (Oy Growth Curves Ab Ltd.) for a period of 48 hours at 28°C, using continuous shaking and OD_600_ measurements every 30 minutes.

#### Oxygen consumption measurement

Oxygen consumption was measured using a FireSting oxygen electrode (Pyro-Science) under constant stirring at a temperature of 28.0 ± 0.2°C in sealed 2 ml bottles. The corresponding cell counts were measured using a CASY Cell Counter, whereas percentage of living cells in the sample were established by flow cytometry with propidium iodide (PI: 100 ng/ml) stained samples. The slope of the oxygen concentration as the function of time in its linear region was calculated and normalized to the number of living cells in the sample.

#### ROS accumulation (DHE) assay

Oxidation of non-fluorescent di-hydroethidium (DHE) to fluorescent ethidium was used to measure ROS accumulation in yeast cells 38. Approximately 5*10^6^ cells from each sample were collected, washed and incubated with DHE solution (2.5 µg/ml in PBS) for 10 min in the dark. After washing samples were re-suspended in PBS buffer and measured using flow cytometry. The relative mean fluorescence measured for the cell population was used for analysis 70.

#### Boiling ethanol extraction of ATP and ATP measurement

ATP extraction was done with flash-frozen cells by adding 0.5 ml preheated (90°C) BES buffer and incubation at 90°C for 3 minutes. After centrifugation, supernatants were stored at -80°C until the measurement. ATP levels were determined by using the ATP detection kit from Invitrogen in a Luminoskan (Thermo Scientific).

#### Cytosolic Ca^2+^ measurements

[Ca^2+^]cyt were measured using yeast strains carrying the vector pYX212 encoding the bioluminescent protein aequorin under the control of a TPI promoter. For analysis of the cellular response to high doses of external Ca^2+^, an equivalent of 6*10^6^ cells was harvested, resuspended in 200 μl SCD containing 4 μM coelenterazine and incubated for 1 h in the dark. After washing cells were measured in a Luminoskan for 10 s and then challenged with high dose of Ca^2+ ^(pump injection of 150 mM Ca^2+^). Kinetics were recorded over 120 s. The luminescence signal was normalized to the OD_600_ of each well and reported in relative luminescence units, normalized to the global maximum value of the ethanol treated control of the respective run for better comparability.

### SUPPLEMENTAL MATERIAL

Click here for supplemental data file.

All supplemental data for this article are also available online at "http://microbialcell.com/researcharticles/the-neuroprotective-steroid-progesterone-promotes-mitochondrial-uncoupling-reduces-cytosolic-calcium-and-augments-stress-resistance-in-yeast-cells/".

## References

[1] Baulieu E, Schumacher M (2000). Progesterone as a neuroactive neurosteroid, with special reference to the effect of progesterone on myelination.. Steroids.

[2] Vagnerova K, Koerner IP, Hurn PD (2008). Gender and the Injured Brain.. Anesth Analg.

[3] Meffre D, Labombarda F, Delespierre B, Chastre A, De Nicola AF, Stein DG, Schumacher M, Guennoun R (2013). Distribution of membrane progesterone receptor alpha in the male mouse and rat brain and its regulation after traumatic brain injury.. Neuroscience.

[4] Si D, Wang H, Wang Q, Zhang C, Sun J, Wang Z, Zhang Z, Zhang Y (2013). Progesterone treatment improves cognitive outcome following experimental traumatic brain injury in rats.. Neurosci Lett.

[5] Soltani Z, Khaksari M, Shahrokhi N, Mohammadi G, Mofid B, Vaziri A, Amiresmaili S (2016). Effect of estrogen and/or progesterone administration on traumatic brain injury-caused brain edema: the changes of aquaporin-4 and interleukin-6.. J Physiol Biochem.

[6] Carswell HV, Anderson NH, Clark JS, Graham D, Jeffs B, Dominiczak AF, Macrae IM (1999). Genetic and gender influences on sensitivity to focal cerebral ischemia in the stroke-prone spontaneously hypertensive rat.. Hypertens Dallas Tex 1979.

[7] Alkayed NJ, Murphy SJ, Traystman RJ, Hurn PD, Miller VM (2000). Neuroprotective effects of female gonadal steroids in reproductively senescent female rats.. Stroke.

[8] Xiao G, Wei J, Yan W, Wang W, Lu Z (2008). Improved outcomes from the administration of progesterone for patients with acute severe traumatic brain injury: a randomized controlled trial.. Crit Care Lond Engl.

[9] Wright DW, Kellermann AL, Hertzberg VS, Clark PL, Frankel M, Goldstein FC, Salomone JP, Dent LL, Harris OA, Ander DS, Lowery DW, Patel MM, Denson DD, Gordon AB, Wald MM, Gupta S, Hoffman SW, Stein DG (2007). ProTECT: A Randomized Clinical Trial of Progesterone for Acute Traumatic Brain Injury.. Ann Emerg Med.

[10] Skolnick BE, Maas AI, Narayan RK, van der Hoop RG, MacAllister T, Ward JD, Nelson NR, Stocchetti N, SYNAPSE Trial Investigators (2014). A clinical trial of progesterone for severe traumatic brain injury.. N Engl J Med.

[11] Lin C, He H, Li Z, Liu Y, Chao H, Ji J, Liu N (2015). Efficacy of progesterone for moderate to severe traumatic brain injury: a meta-analysis of randomized clinical trials.. Sci Rep.

[12] Zeng Y, Zhang Y, Ma J, Xu J (2015). Progesterone for Acute Traumatic Brain Injury: A Systematic Review of Randomized Controlled Trials.. PloS One.

[13] Madeo F, Fröhlich E, Fröhlich KU (1997). A yeast mutant showing diagnostic markers of early and late apoptosis.. J Cell Biol.

[14] Madeo F, Fröhlich E, Ligr M, Grey M, Sigrist SJ, Wolf DH, Fröhlich KU (1999). Oxygen stress: a regulator of apoptosis in yeast.. J Cell Biol.

[15] Madeo F, Herker E, Maldener C, Wissing S, Lächelt S, Herlan M, Fehr M, Lauber K, Sigrist SJ, Wesselborg S, Fröhlich KU (2002). A caspase-related protease regulates apoptosis in yeast.. Mol Cell.

[16] Herker E, Jungwirth H, Lehmann KA, Maldener C, Fröhlich K-U, Wissing S, Büttner S, Fehr M, Sigrist S, Madeo F (2004). Chronological aging leads to apoptosis in yeast.. J Cell Biol.

[17] Wissing S, Ludovico P, Herker E, Büttner S, Engelhardt SM, Decker T, Link A, Proksch A, Rodrigues F, Corte-Real M, Fröhlich K-U, Manns J, Candé C, Sigrist SJ, Kroemer G, Madeo F (2004). An AIF orthologue regulates apoptosis in yeast.. J Cell Biol.

[18] Büttner S, Ruli D, Vögtle F-N, Galluzzi L, Moitzi B, Eisenberg T, Kepp O, Habernig L, Carmona-Gutierrez D, Rockenfeller P, Laun P, Breitenbach M, Khoury C, Fröhlich K-U, Rechberger G, Meisinger C, Kroemer G, Madeo F (2011). A yeast BH3-only protein mediates the mitochondrial pathway of apoptosis.. EMBO J.

[19] Galluzzi L, Kepp O, Kroemer G (2016). Mitochondrial regulation of cell death: a phylogenetically conserved control.. Microb Cell.

[20] Büttner S, Habernig L, Broeskamp F, Ruli D, Vögtle FN, Vlachos M, Macchi F, Küttner V, Carmona-Gutierrez D, Eisenberg T, Ring J, Markaki M, Taskin AA, Benke S, Ruckenstuhl C, Braun R, Van den Haute C, Bammens T, van der Perren A, Fröhlich K-U, Winderickx J, Kroemer G, Baekelandt V, Tavernarakis N, Kovacs GG, Dengjel J, Meisinger C, Sigrist SJ, Madeo F (2013). Endonuclease G mediates α-synuclein cytotoxicity during Parkinson’s disease.. EMBO J.

[21] Büttner S, Broeskamp F, Sommer C, Markaki M, Habernig L, Alavian-Ghavanini A, Carmona-Gutierrez D, Eisenberg T, Michael E, Kroemer G, Tavernarakis N, Sigrist SJ, Madeo F (2014). Spermidine protects against α-synuclein neurotoxicity.. Cell Cycle Georget Tex.

[22] Heinisch JJ, Brandt R (2016). Signaling pathways and posttranslational modifications of tau in Alzheimer’s disease: the humanization of yeast cells.. Microb Cell.

[23] Menezes R, Tenreiro S, Macedo D, Santos C, Outeiro T (2015). From the baker to the bedside: yeast models of Parkinson’s disease.. Microb Cell.

[24] Amen T, Kaganovich D (2016). Yeast screening platform identifies FDA-approved drugs that reduce Aβ oligomerization.. Microb Cell.

[25] Shrestha A, Megeney L (2015). Yeast proteinopathy models: a robust tool for deciphering the basis of neurodegeneration.. Microb Cell.

[26] Bond M, Brown R, Rallis C, Bahler J, Mole S (2015). A central role for TOR signalling in a yeast model for juvenile CLN3 disease.. Microb Cell.

[27] Carmona-Gutierrez D, Hughes AL, Madeo F, Ruckenstuhl C (2016). The crucial impact of lysosomes in aging and longevity.. Ageing Res Rev.

[28] Raghupathi R (2004). Cell death mechanisms following traumatic brain injury.. Brain Pathol Zurich Switz.

[29] Cheng G, Kong R, Zhang L, Zhang J (2012). Mitochondria in traumatic brain injury and mitochondrial-targeted multipotential therapeutic strategies: Mitochondria in traumatic brain injury.. Br J Pharmacol.

[30] Ludovico P, Sousa MJ, Silva MT, Leão C, Côrte-Real M (2001). Saccharomyces cerevisiae commits to a programmed cell death process in response to acetic acid.. Microbiol Read Engl.

[31] Rockenfeller P, Ring J, Muschett V, Beranek A, Buettner S, Carmona-Gutierrez D, Eisenberg T, Khoury C, Rechberger G, Kohlwein SD, Kroemer G, Madeo F (2010). Fatty acids trigger mitochondrion-dependent necrosis.. Cell Cycle Georget Tex.

[32] Eisenberg T, Carmona-Gutierrez D, Büttner S, Tavernarakis N, Madeo F (2010). Necrosis in yeast.. Apoptosis Int J Program Cell Death.

[33] Carmona-Gutiérrez D, Bauer MA, Ring J, Knauer H, Eisenberg T, Büttner S, Ruckenstuhl C, Reisenbichler A, Magnes C, Rechberger GN, Birner-Gruenberger R, Jungwirth H, Fröhlich K-U, Sinner F, Kroemer G, Madeo F (2011). The propeptide of yeast cathepsin D inhibits programmed necrosis.. Cell Death Dis.

[34] Braun RJ, Sommer C, Carmona-Gutierrez D, Khoury CM, Ring J, Büttner S, Madeo F (2011). Neurotoxic 43-kDa TAR DNA-binding protein (TDP-43) triggers mitochondrion-dependent programmed cell death in yeast.. J Biol Chem.

[35] Holmström KM, Finkel T (2014). Cellular mechanisms and physiological consequences of redox-dependent signalling.. Nat Rev Mol Cell Biol.

[36] Braun RJ, Sommer C, Leibiger C, Gentier RJG, Dumit VI, Paduch K, Eisenberg T, Habernig L, Trausinger G, Magnes C, Pieber T, Sinner F, Dengjel J, van Leeuwen FW, Kroemer G, Madeo F (2015). Accumulation of Basic Amino Acids at Mitochondria Dictates the Cytotoxicity of Aberrant Ubiquitin.. Cell Rep.

[37] Braun R, Sommer C, Leibiger C, Gentier R, Dumit V, Paduch K, Eisenberg T, Habernig L, Trausinger G, Magnes C, Pieber T, Sinner F, Dengjel J, van Leeuwen F, Kroemer G, Madeo F (2015). Modeling non-hereditary mechanisms of Alzheimer disease during apoptosis in yeast.. Microb Cell.

[38] Ruckenstuhl C, Büttner S, Carmona-Gutierrez D, Eisenberg T, Kroemer G, Sigrist SJ, Fröhlich K-U, Madeo F (2009). The Warburg Effect Suppresses Oxidative Stress Induced Apoptosis in a Yeast Model for Cancer.. PLoS ONE.

[39] Hand RA, Jia N, Bard M, Craven RJ (2003). Saccharomyces cerevisiae Dap1p, a novel DNA damage response protein related to the mammalian membrane-associated progesterone receptor.. Eukaryot Cell.

[40] Gregor A, Kocyłowski M, Kostrzewska E (1986). Evaluation of the diagnostic usefulness of determining porphobilinogen deaminase activity in the erythrocytes in patients with acute intermittent porphyria and in carriers of the gene of this type of porphyria.. Przegl Lek.

[41] Tubiana N, Mishal Z, le Caer F, Seigneurin JM, Berthoix Y, Martin PM, Carcassonne Y (1986). Quantification of oestradiol binding at the surface of human lymphocytes by flow cytofluorimetry.. Br J Cancer.

[42] Roussel D, Harding M, Runswick MJ, Walker JE, Brand MD (2002). Does any yeast mitochondrial carrier have a native uncoupling protein function?. J Bioenerg Biomembr.

[43] Barros MH, Bandy B, Tahara EB, Kowaltowski AJ (2004). Higher respiratory activity decreases mitochondrial reactive oxygen release and increases life span in Saccharomyces cerevisiae.. J Biol Chem.

[44] Skulachev VP (1998). Uncoupling: new approaches to an old problem of bioenergetics.. Biochim Biophys Acta BBA - Bioenerg.

[45] Mookerjee SA, Divakaruni AS, Jastroch M, Brand MD (2010). Mitochondrial uncoupling and lifespan.. Mech Ageing Dev.

[46] Amara CE, Shankland EG, Jubrias SA, Marcinek DJ, Kushmerick MJ, Conley KE (2007). Mild mitochondrial uncoupling impacts cellular aging in human muscles in vivo.. Proc Natl Acad Sci.

[47] Rodriguez AM, Monjo M, Roca P, Palou A (2002). Opposite actions of testosterone and progesterone on UCP1 mRNA expression in cultured brown adipocytes.. Cell Mol Life Sci CMLS.

[48] Wade R, Jones Jr HW (1956). Effect of progesterone on mitochondrial adenosinetriphospatase.. JBC.

[49] Liu W (2012). Control of Calcium in Yeast Cells.. In: Introduction to Modeling Biological Cellular Control Systems..

[50] Ren ZW (1992). Radiofrequency ablation of left-sided atrioventricular accessory tract to treat supraventricular tachycardia.. Zhonghua Xin Xue Guan Bing Za Zhi.

[51] Romarowski A, Sánchez-Cárdenas C, Ramírez-Gómez HV, Puga Molina L del C, Treviño CL, Hernández-Cruz A, Darszon A, Buffone MG (2016). A Specific Transitory Increase in Intracellular Calcium Induced by Progesterone Promotes Acrosomal Exocytosis in Mouse Sperm.. Biol Reprod.

[52] Li L-F, Xiang C, Zhu Y-B, Qin K-R (2014). Modeling of progesterone-induced intracellular calcium signaling in human spermatozoa.. J Theor Biol.

[53] Bonaccorsi L (1998). Progesterone-stimulated intracellular calcium increase in human spermatozoa is protein kinase C-independent.. Mol Hum Reprod.

[54] Miller MR, Mannowetz N, Iavarone AT, Safavi R, Gracheva EO, Smith JF, Hill RZ, Bautista DM, Kirichok Y, Lishko PV (2016). Unconventional endocannabinoid signaling governs sperm activation via the sex hormone progesterone.. Science.

[55] Batra S (1986). Effect of estrogen and progesterone treatment on calcium uptake by the myometrium and smooth muscle of the lower urinary tract.. Eur J Pharmacol.

[56] Barbagallo M, Dominguez LJ, Licata G, Shan J, Bing L, Karpinski E, Pang PKT, Resnick LM (2001). Vascular Effects of Progesterone : Role of Cellular Calcium Regulation.. Hypertens Dallas Tex 1979.

[57] He Y, Gao Q, Han B, Zhu X, Zhu D, Tao J, Chen J, Xu Z (2016). Progesterone suppressed vasoconstriction in human umbilical vein via reducing calcium entry.. Steroids.

[58] Wu Z, Shen W (2010). Progesterone inhibits L-type calcium currents in gallbladder smooth muscle cells.. J Gastroenterol Hepatol.

[59] Luoma JI, Kelley BG, Mermelstein PG (2011). Progesterone inhibition of voltage-gated calcium channels is a potential neuroprotective mechanism against excitotoxicity.. Steroids.

[60] Luoma JI, Stern CM, Mermelstein PG (2012). Progesterone inhibition of neuronal calcium signaling underlies aspects of progesterone-mediated neuroprotection.. J Steroid Biochem Mol Biol.

[61] Cai W, Zhu Y, Furuya K, Li Z, Sokabe M, Chen L (2008). Two different molecular mechanisms underlying progesterone neuroprotection against ischemic brain damage.. Neuropharmacology.

[62] Brotfain E, Gruenbaum SE, Boyko M, Kutz R, Zlotnik A, Klein M (2016). Neuroprotection by Estrogen and Progesterone in Traumatic Brain Injury and Spinal Cord Injury.. Curr Neuropharmacol.

[63] Wei J, Xiao G (2013). The neuroprotective effects of progesterone on traumatic brain injury: current status and future prospects.. Acta Pharmacol Sin.

[64] Pascual JL, Murcy MA, Li S, Gong W, Eisenstadt R, Kumasaka K, Sims C, Smith DH, Browne K, Allen S, Baren J (2013). Neuroprotective effects of progesterone in traumatic brain injury: blunted in vivo neutrophil activation at the blood-brain barrier.. Am J Surg.

[65] Li Z, Wang B, Kan Z, Zhang B, Yang Z, Chen J, Wang D, Wei H, Zhang J, Jiang R (2012). Progesterone increases circulating endothelial progenitor cells and induces neural regeneration after traumatic brain injury in aged rats.. J Neurotrauma.

[66] Webster KM, Wright DK, Sun M, Semple BD, Ozturk E, Stein DG, O’Brien TJ, Shultz SR (2015). Progesterone treatment reduces neuroinflammation, oxidative stress and brain damage and improves long-term outcomes in a rat model of repeated mild traumatic brain injury.. J Neuroinflammation.

[67] Gueldener U, Heinisch J, Koehler GJ, Voss D, Hegemann JH (2002). A second set of loxP marker cassettes for Cre-mediated multiple gene knockouts in budding yeast.. Nucleic Acids Res.

[68] Güldener U, Heck S, Fielder T, Beinhauer J, Hegemann JH (1996). A new efficient gene disruption cassette for repeated use in budding yeast.. Nucleic Acids Res.

[69] Loewith R, Jacinto E, Wullschleger S, Lorberg A, Crespo JL, Bonenfant D, Oppliger W, Jenoe P, Hall MN (2002). Two TOR complexes, only one of which is rapamycin sensitive, have distinct roles in cell growth control.. Mol Cell.

[70] Kainz K, Tadic J, Zimmermann A, Pendl  T,  Carmona-Gutierrez  D, Ruckenstuhl C, Eisenberg T, Madeo F (2017). Methods to Assess Autophagy and Chronological Aging in Yeast.. In: Methods in Enzymology.

