# Supplementary material for: The neuroprotective steroid progesterone promotes mitochondrial uncoupling, reduces cytosolic calcium and augments stress resistance in yeast cells
Source: Microb Cell. 2017 May 31;4(6):191–9. doi: 10.15698/mic2017.06.577 (PMC5473691; doi:10.15698/mic2017.06.577)
Supplement: Supplementary file 1 [file mic-04-191-s01.pdf]

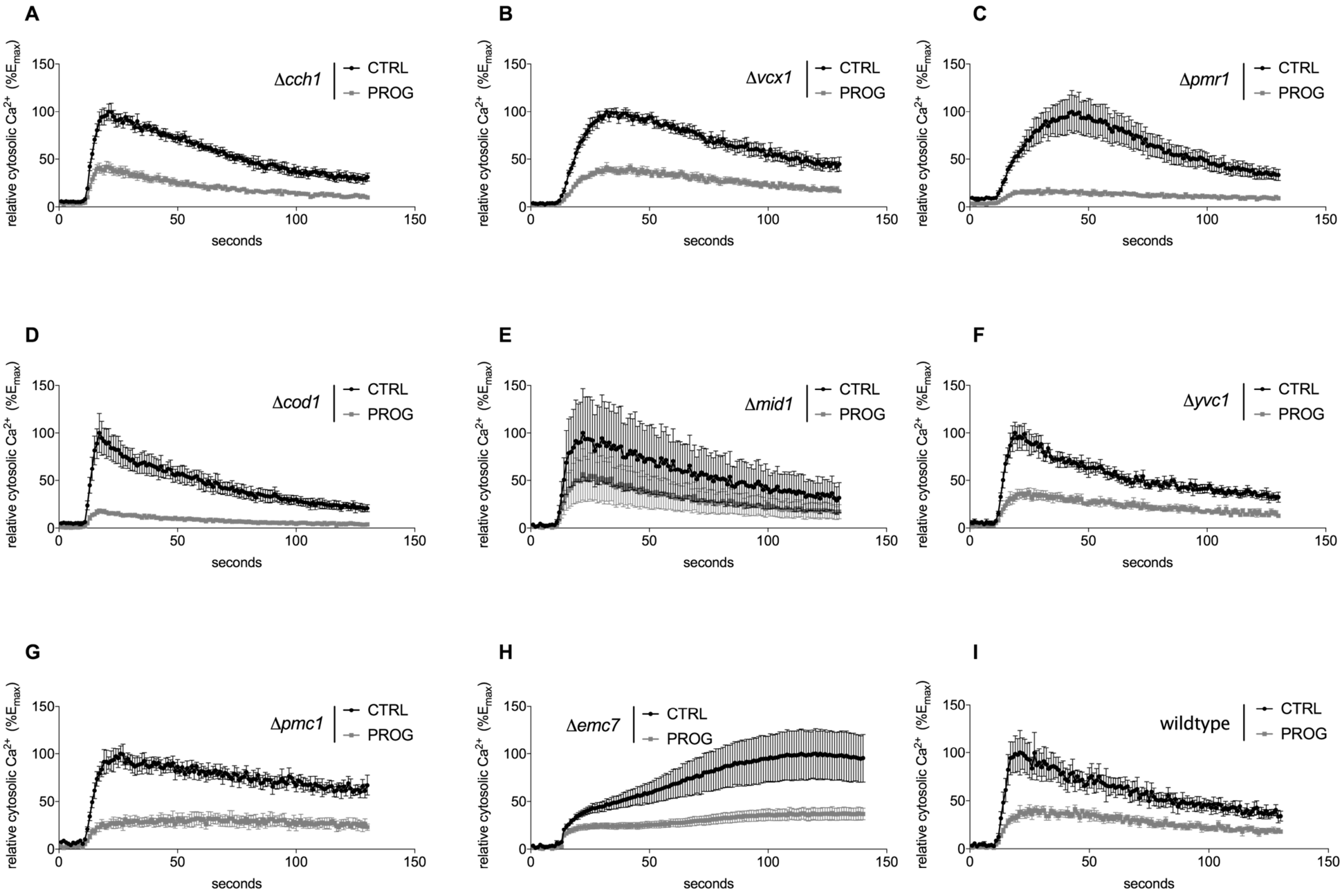

Supplemental FIGURE 1: Modulation of Ca^{2+} homeostasis by progesterone in yeast is independent of known Ca^{2+} channels. Cells were treated with progesterone (10 µg/ml) and challenged with high doses of Ca^{2+} (150 mM). Intake of Ca^{2+} as well as Ca^{2+} -clearance in the cytosol to its basal level were measured in indicated single gene deletion mutants **(A-H)** as well as the corresponding wild type strain **(I)** (n = 3-4± SEM) Prog = progesterone, ctrl = control.
